# Supplementary material for: Designing a Novel Monitoring Approach for the Effects of Space Travel on Astronauts’ Health
Source: Life (Basel). 2023 Feb 18;13(2):576. doi: 10.3390/life13020576 (PMC9964234; doi:10.3390/life13020576)
Supplement: Supplementary file 1 [file life-13-00576-s001.zip › Supplementary Figure S1.pdf]

Supplementary Figure S1. Protein-protein interaction (**A**), disease-gene (**B**), drug-gene (**C**) and miRNA-gene (**D**) networks for the 218 differentially expressed genes (DEGs) between Pre-flight and In-flight gene expression datasets collected from 10 astronauts (8 males and 2 females). Nodes with higher degrees of connectivity were shown in larger size and darker color. Genes identified for the rapid RT^2-PCR assay kit were labelled in the networks. Genes *CCNC*, *CTNNBIP1*, *EIF4E2* and *RRN3* are not present in the disease-gene network; genes *BIRC2*, *CCNC*, *CHMP4C*, *CTNNBIP1*, *EIF4E2*, *IL7*, *KIDINS220*, *MAGT1*, *PIKFYVE*, *RRN3*, *SOS2*, *SPON2*, *THBS3*, *XAF1* and *ZNF606* are not present in the drug-gene network; and genes *CRHR1*, *DPP4*, *GPT* and *SPON2* are not present in the miRNA-gene network, respectively.

A

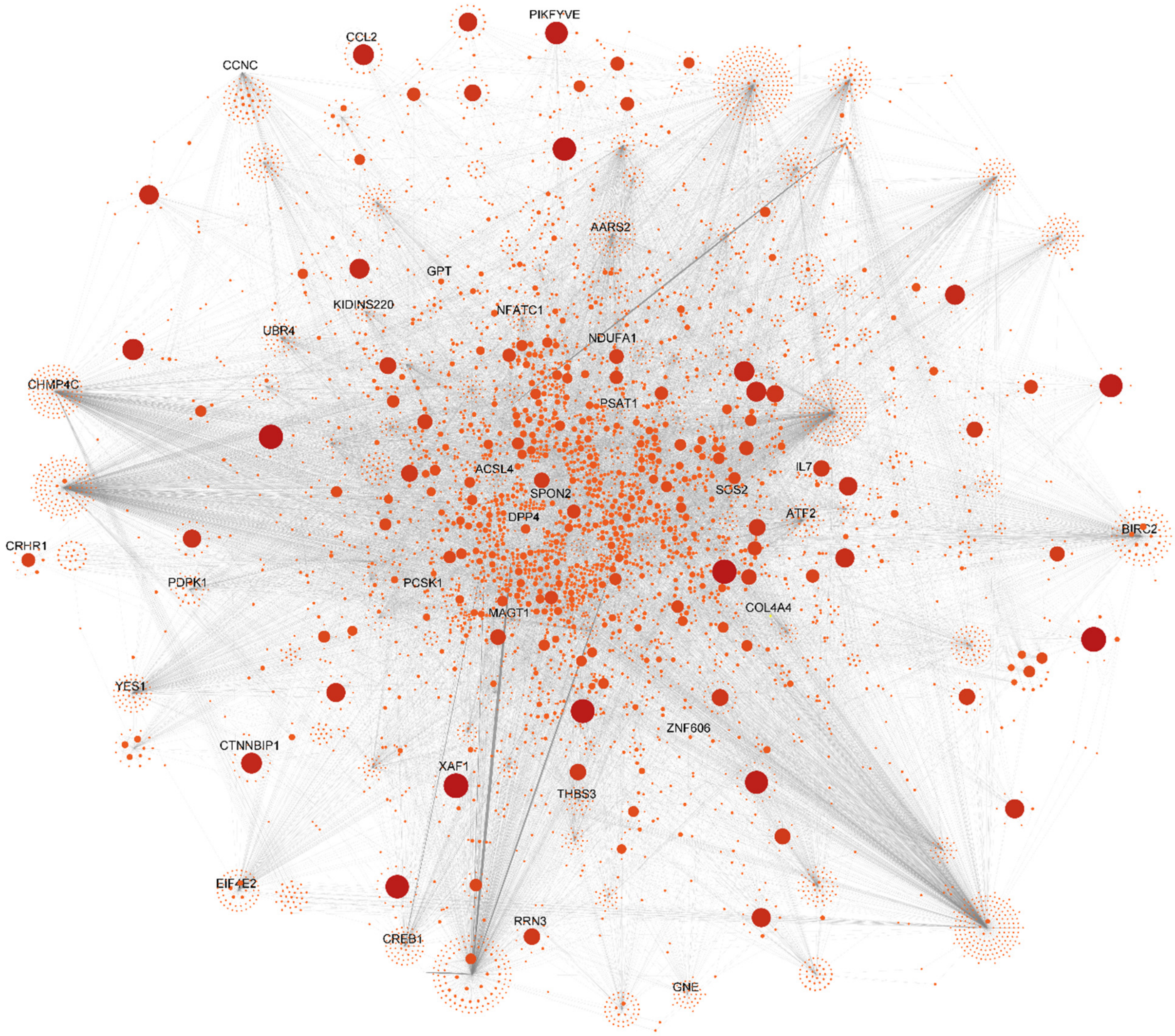

B

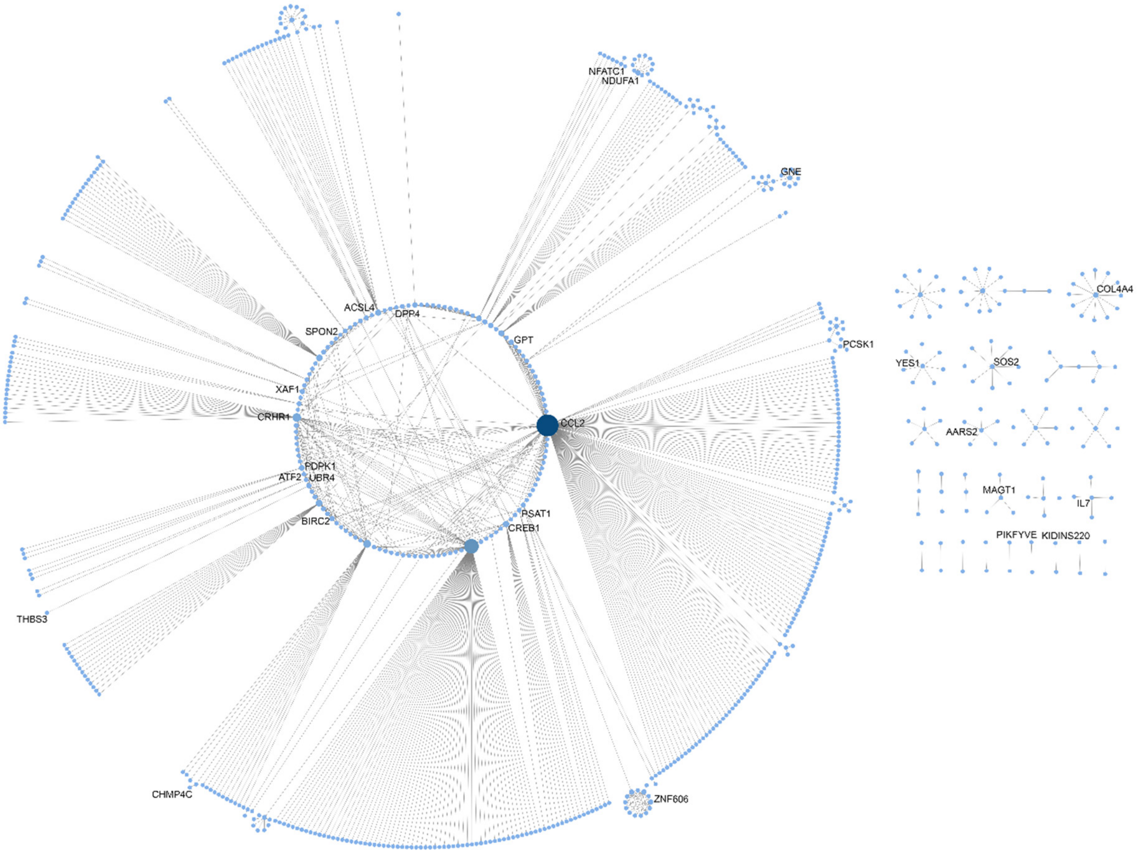
NFATC1
NDUFA1
GNE
ACSL4
DPP4
SPON2
GPT
PCSK1
XAF1
CRHR1
CCL2
PDPK1
ATF2
UBR4
BIRC2
PSAT1
CREB1
THBS3
CHMP4C
ZNF606
COL4A4
YES1
SOS2
AARS2
MAGT1
IL7
PIKFYVE
KIDINS220

C

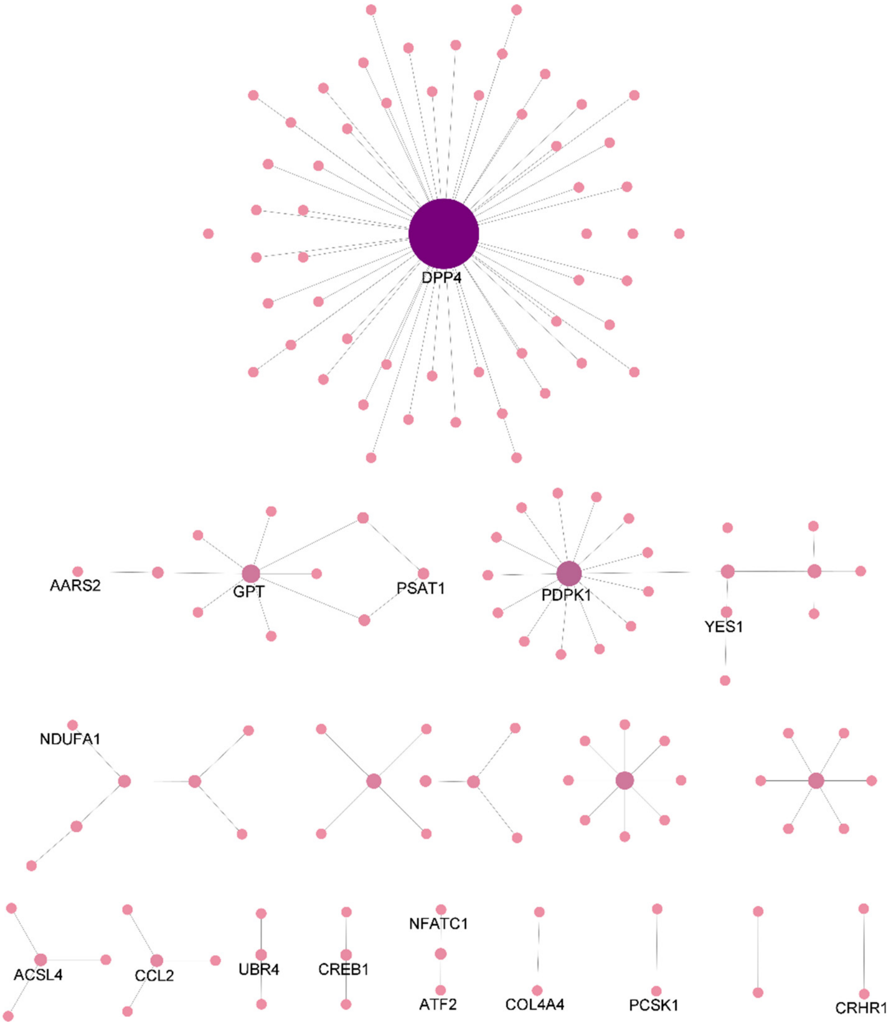
DPP4
AARS2
GPT
PSAT1
PDPK1
YES1
NDUFA1
ACSL4
CCL2
UBR4
CREB1
NFATC1
ATF2
COL4A4
PCSK1
CRHR1

D

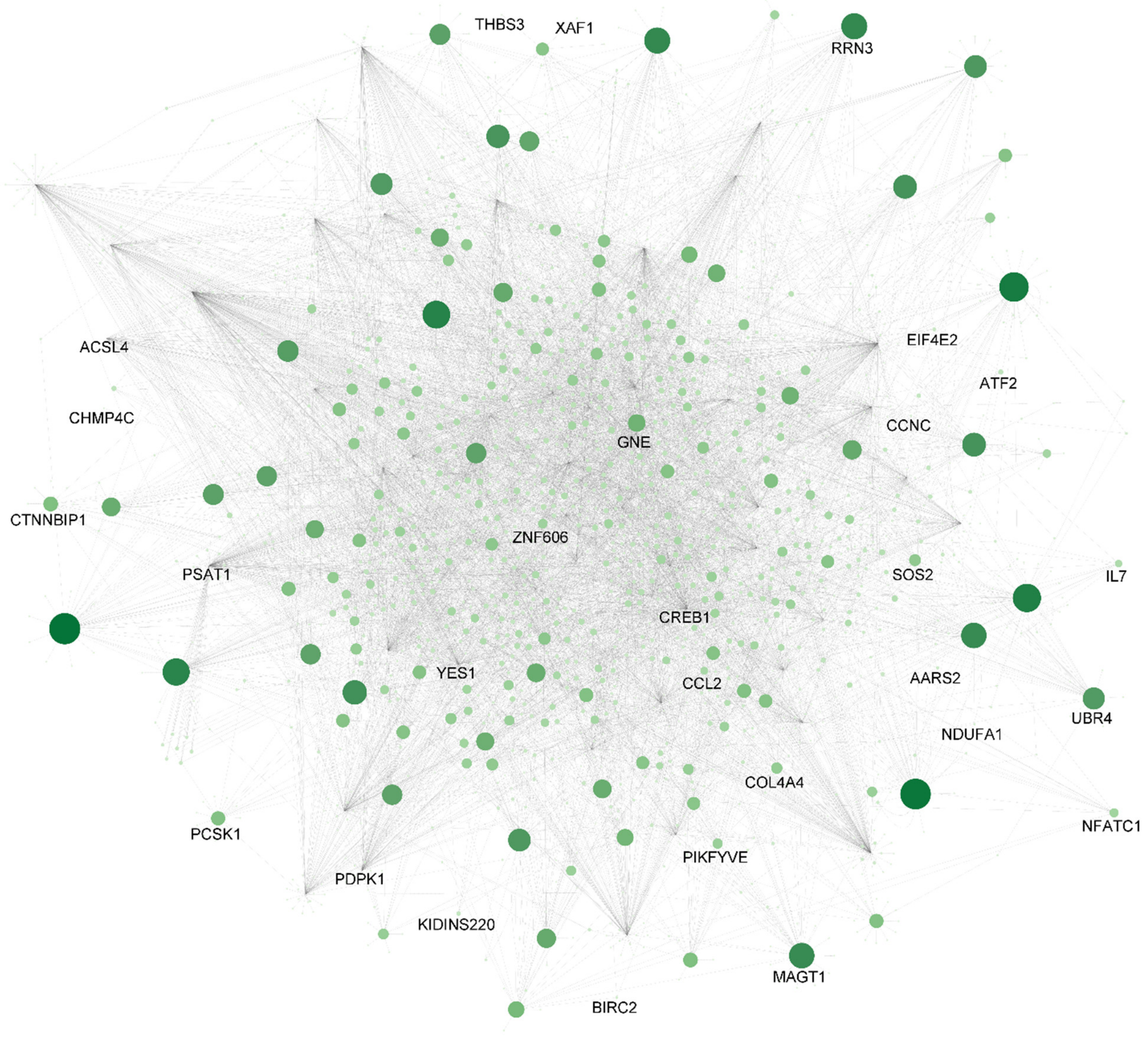
THBS3
XAF1
RRN3
ACSL4
EIF4E2
ATF2
CHMP4C
CCNC
GNE
CTNNBIP1
ZNF606
PSAT1
SOS2
IL7
CREB1
YES1
CCL2
AARS2
UBR4
NDUFA1
COL4A4
NFATC1
PCSK1
PIKFYVE
PDPK1
KIDINS220
MAGT1
BIRC2
